# Changes in the Diversity of Human Skin Microbiota to Cosmetic Serum Containing Prebiotics: Results from a Randomized Controlled Trial

**DOI:** 10.3390/jpm10030091

**Published:** 2020-08-17

**Authors:** Ki-Bae Hong, Yang Hee Hong, Eun Young Jung, Kyungae Jo, Hyung Joo Suh

**Affiliations:** 1 Department of Integrated Biomedical and Life Sciences, Graduate School, Korea University, Seoul 02841, Korea; kibae.hong@gmail.com; 2 Department of Beauty Art, Suwon Women’s University, Suwon 16632, Korea; hongyh@swc.ac.kr; 3 Department of Home Economic Education, Jeonju University, Jeonju 55069, Korea; jjjj@jj.ac.kr; 4 BK21Plus, College of Health Science, Korea University, Seoul 02841, Korea; kyungae11@korea.ac.kr

**Keywords:** prebiotics, GOS, skin microbiota, *Staphylococcus aureus*

## Abstract

Prebiotic treatment may rebalance the skin microbiota by regulating the growth of harmful and beneficial microorganisms. In this randomized, double-blind, placebo-controlled clinical trial (N = 60), we evaluated the effects of a cosmetic serum containing galacto-oligosaccharides (GOS) on the balance of the skin microbiota by measuring various skin parameters. The skin water-holding capacity between the control (ND) and experimental (NF) groups was significantly different after 8 weeks of serum treatment (*p* < 0.05). Similarly, changes in transepidermal water loss (TEWL) and the erythema index in the ND and NF groups were significantly different (*p* < 0.05). Furthermore, the wrinkle depth and *Staphylococcus aureus* population decreased in the NF group compared with those in the ND group (*p* < 0.05). The mean form factor, Shannon index, and *Pediococcus* population were significantly increased in the post-NF group compared with those in the post-ND group (*p* < 0.05). Finally, in the ND group, water-holding capacity was positively correlated with *Enhydrobacter*, whereas *Enterobacteriaceae* was negatively correlated with TEWL in the NF group. These results suggest that GOS inhibit the growth of harmful skin microbes and increase the population of beneficial microbes.

## 1. Introduction

The skin acts as a barrier that defends against potential hazards and toxins from the surrounding environment [1]. The skin is the point of contact between the external and internal environment and allows the growth of various microorganisms, including fungi, bacteria, viruses, and small larvae [2]. The skin microbiota plays a major role in the suppression of pathogenic species and regulation of skin proteins, free fatty acids, and lipid-rich substances [3]. The skin combines physical and biological factors to create a microbial habitat, and factors that affect symbiosis between humans and microorganisms may impair skin barrier functions, leading to infections [4].

The skin microbiota produces various metabolites, such as aureusimine A and B, which directly induce differential gene expression in human keratinocytes [5,6,7]. The functions of these metabolites are reported to be involved in the expression of the virulence factor gene in *S. aureus*, inhibition of protease in eukaryotic cells, and interspecies bacterial communication [7]. It is necessary to use antimicrobial agents that inhibit the growth of harmful bacteria, such as *Propionibacterium acnes*, or create an environment that favors the growth of beneficial microorganisms, such as *Staphylococcus epidermis*. Prebiotic treatment can cause specific changes in the microbial diversity and colonization that could rebalance the skin microbiota [8]. Several attempts have been directed towards the use of prebiotic cosmetics to balance the composition of skin microbiota; however, the effects of galacto-oligosaccharides (GOS), a prebiotic, on skin microbiota diversity are still unknown [8,9].

GOS are indigestible food components that could improve the health of the host by selectively increasing the population of beneficial microorganisms [10]. In our previous study [11], oral administration of GOS (2 g/day) decreased the trans-epidermal water loss (TEWL), increased the water-holding capacity, and reduced the area of wrinkles on the skin. Further, GOS cause changes in intestinal microbiota and the mucosal immune system, which influence the skin. In addition, like prebiotics, the use of cosmetic formulation containing sugar and beta-glucan has been reported to improve various human skin conditions such as skin dehydration and wrinkle formation. However, whether GOS can have such beneficial effects on skin conditions remains unknown [12,13].

In the present clinical trial, a cosmetic serum containing GOS was directly applied to the facial skin to investigate the changes in the microorganisms on the alar crease (side of the nose). We evaluated the 8-week treatment with GOS-containing cosmetic serum is effective in improving various skin parameters and the population of beneficial skin microbes.

## 2. Materials and Methods

### 2.1. Cosmetic Serum Formulation

The cosmetic serum (Formulation NF) was prepared using commercially available PEG-60 hydrogenated castor oil (0.3%), cetyl ethylhexanoate (0.5%), arginine (0.17%), hydroxyethylcellulose (0.1%), carbomer #941 (0.07%), carbomer #940 (0.1%), ethylhexylglycerin (0.05%), phenoxyethanol (0.4%), disodium ethylenediaminetetraacetic acid (EDTA, 0.03%), GOS (7.0%), and deionized water (to reach 100%). Serum without GOS (Formulation ND) was used as control. The serum samples were mixed at room temperature. Formulation ND and NF were obtained from Phytacoid Inc., Suwon, Korea. GOS used in the cosmetic serum was donated by Neo Crema Co., Ltd. (Seoul, Korea).

### 2.2. Subjects and Sampling

The subjects of this study were healthy women in their 40s and 60s, and all subjects signed a clinical trial agreement after receiving a detailed explanation on the purpose and contents of the study. There were no dropouts, and a total of 60 measurement results were used for statistical analysis. None of the subjects had a history of skin infections, skin diseases, or other chronic diseases and were in good health and did not receive antibiotics or antifungal agents for more than 1 month. According to Fitzpatrick classification, the subjects were classified as skin types II and III [14] and were instructed to disuse hygiene products such as cosmetics, moisturizers, and antiperspirant deodorants for 12 h prior to sampling. In a randomized controlled trial, 60 women were screened and received either the placebo (formulation ND; N = 30; the median age: 50.33; 40s: 19; 50s: 5; 60s: 6) or cosmetic serum (formulation NF; N = 30; the median age: 50.43; 40s: 20; 50s: 5; 60s: 5) for 8 weeks. All subjects applied a total of 1.0 mL of the sample to their faces twice a day (0.5 mL in the morning and at night) for 8 weeks, and skin properties were evaluated. For skin microbiota analysis, subjects were investigated by the researcher who proceeded the study and collected samples. Subjects who used medicinal soaps and facial cleanser 6 h before obtaining analytical samples (n = 32), and those who did not wish to analyze their composition of skin microbiota (n = 12) were not included in the experiment. Additionally, only the subjects who provided written consent were analyzed for the skin microbiota and were randomly divided into groups (n = 16). Skin samples for skin microbiota analysis were obtained from the alar crease (side of the nose) with a sterile cotton swab. The head of each swab was cut-off and stored at 4 °C until DNA extraction. The study was approved by the Jeonju University Institutional Review Board (jjIRB-190115-HR-2019-0106) in Korea and was conducted in accordance with the ethical standards of 1964 Declaration of Helsinki.

### 2.3. Skin Assessments

The surface and TEWL of the skin were measured using the Corneometer CM825 (Courage and Khazaka electronic GmbH, Cologne, Germany) and Tewameter TM300 (Courage and Khazaka electronic GmbH, Cologne, Germany). Melanin index and erythema index of the skin were measured using the Mexameter MX18 (Courage and Khazaka electronic GmbH, Cologne, Germany). To measure wrinkles from the corner of the eye (Crow’s feet), the skin replicas were obtained before and after the experiment using the silicon solution Silflo (Flexico Ltd., London, England). All simulated plates were analyzed for six different wrinkle parameters: total wrinkle area, percent of wrinkle area, number of wrinkles, total length, wrinkle depth, and mean form factor, using the Visioline VL650 (Courage and Khazaka electronic GmbH, Cologne, Germany).

### 2.4. DNA Extraction, Amplification, and Sequencing

DNA from the skin microbiota was extracted from the cotton swab with i-genomic Clinic DNA Extraction Kit (iNtRON Biotech, Sung-Nam, Korea) according to the manufacturer’s protocol. The 16S rRNA amplicon libraries were constructed by slightly modifying the protocol of Illumina 16S V3~V4 Amplicon (Illumina, San Diego, CA, USA). The 16S rRNA V3~V4 regions were amplified by first polymerase chain reaction (PCR-I) as follows: 2 min at 95 °C, 25 cycles of 30 s at 95 °C, 30 s at 55 °C, and 30 s at 72 °C, and 10 min at 72 °C. The PCR products were purified using the Agencourt AMPure XP Beads (Beckman Coulter, Inc., Pasadena, CA, USA) and then quantified. A second PCR (PCR-II) was conducted using a quality-controlled PCR product as a template and the Illumina dual-index adapter sequences. The PCR-II cycling conditions were as follows: 3 min at 95 °C, 8 cycles of 30 s at 95 °C, 30 s at 55 °C, and 30 s at 72 °C, and 5 min at 72 °C. Next, amplicons were purified with Agencourt AMPure XP Beads (Beckman Coulter, Inc., Pasadena, CA, USA), and their quality and quantity were assessed with GX caliper HS DNA 1000 (PerkinElmer, Waltham, MA, USA) and the NanoQuant pro200 instrument (Tecan, Mannedorf, Switzerland). The final amplicon libraries were sequenced on an Illumina MiSeq using the v3 600 cycle kit and 301 base-paired end chemistry.

### 2.5. Bioinformatic Analysis Using 16S rRNA Sequences

The sequence data were analyzed using the QIIME 2 software [15]. Prior to sequencing, quality filtering was performed for terminal trimming and to remove low-quality and non-target sequences (<0.2 kb) and ambiguous reads. To eliminate homopolymers, the sequence error rate was minimized using the QIIME pipeline [16]. Representative sequence sets were selected and clustered using UCLUST at a similarity level of 97.0% into OTUs and were then analyzed. Processed sequences were aligned with PyNAST using Greengenes Database (gg_97_otus_4-feb2011.fasta) as a reference [17], and taxonomy was assigned using the ribosomal database project classifier [18] with a minimum confidence score of 0.8. Richness, Shannon Index, and ACE index were included in the alpha-diversity analysis using MOTHUR [19]. Beta-diversity within and between groups was analyzed using UniFrac metric distances. These results were analyzed with PCoA [20].

### 2.6. Statistical Analysis

Data are presented as mean ± standard error. Data were analyzed using one-way ANOVA with Tukey’s post-hoc test using SPSS (v16.0, Chicago, IL, USA). A comparison between two groups was performed using Student’s t-test. Correlation between skin microbial composition (OTU abundance) and the mean of skin parameters in the same group was confirmed using Pearson’s correlation (SPSS version 19.0). To analyze differences in alpha-diversity and relative abundance in bacterial genera, Kruskal–Wallis test was performed. The differences in beta-diversity were analyzed with ANOSIM and permutational multivariate analysis of variance (PERMANOVA) tests. A *p*-value < 0.05 was considered significant.

## 3. Results

### 3.1. Changes in the Water Holding Capacity of the Skin Surface and TEWL by Prebiotics-Containing Cosmetic Serum Treatment

**Figure 1:**
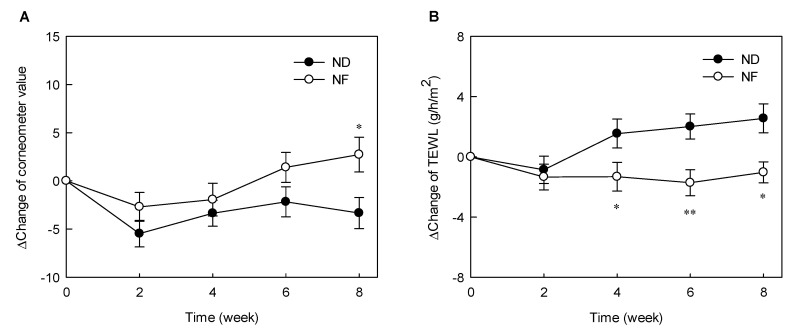
Effects of serum cosmetics containing galacto-oligosaccharides (GOS) on water holding capacity (**A**) and transepidermal water loss (TEWL) (**B**) from baseline after 8 weeks of treatment in healthy adults. The asterisks indicate significant differences (* *p* < 0.05 and ** *p* < 0.01) between the NF and the ND groups at the indicated week. The p of two-sided independent t-test method was displayed, and all data are reported as mean ± standard error of the mean. ND (control serum); NF (cosmetic serum containing GOS).

The delta-values representing of changes in the Corneometer value relative to the initial value are shown in Figure 1. The water-holding capacity was proportional to the increase in the Corneometer values. The values of the control (ND) and experimental (NF) groups at week 0 were 73.26 AU and 69.20 AU, respectively. The skin water-holding capacity decreased gradually after initiating the experimental period but increased slightly after 4 weeks. The skin water-holding capacity of the NF group was significantly higher after 8 weeks of treatment than that of the ND group (*p* < 0.05) (Figure 1A). TEWL refers to the moisture evaporating from the skin and is used as an indicator of the skin barrier function in the stratum corneum. The lower the TEWL, the lower the water loss and the better the skin’s defense ability. The TEWL values of the subjects at the start of the experiment were 13.84 g/h/m^2^ and 14.40 g/h/m^2^ in the ND and NF groups, respectively. The initial TEWL value in the ND group was significantly lower than that at 6 to 8 weeks. Moreover, the delta-values, indicating the change in TEWL in the NF groups, were significantly lower between weeks 4 and 8 than those in the ND group (Figure 1B). These results indicate that GOS can help maintain skin barrier function.

### 3.2. Changes in Skin Pigments by Prebiotics-Containing Cosmetic Serum Treatment

**Figure 2:**
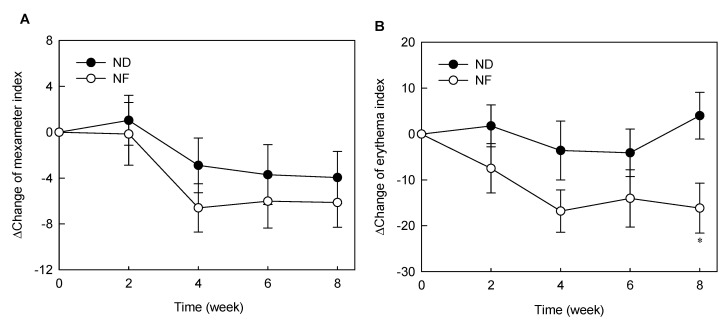
Effects of serum cosmetics containing GOS on melanin index (**A**) and erythema index (**B**) from baseline after 8 weeks of treatment in healthy adults. The asterisks indicate significant differences (* *p* < 0.05) between the NF and the ND groups at the indicated week. The p of two-sided independent t-test method was displayed, and all data are reported as mean ± standard error of the mean. ND (control serum); NF (cosmetic serum containing GOS).

Melanin and erythema pigments were measured during the GOS-containing cosmetic serum treatment period (Figure 2). The melanin pigment decreased during the experimental period in both ND and NF groups. The melanin index of the ND group decreased after 2 weeks, but the difference was not statistically significant. There were also no significant differences between the delta values between the two groups (Figure 2A). Further, the erythema index of the NF group was not significantly changed during the experimental period compared with that of the ND group but was statistically significantly decreased after 4 weeks of treatment compared to the base line (week 0). Figure 2B shows the difference in delta-values between the two groups. The difference of the erythema index between NF and ND groups was statistically significant at week 8 (*p* < 0.05). GOS-containing cosmetic serum was effective in reducing the erythema index but had no significant effect on the melanin pigment.

### 3.3. Changes in the Skin Wrinkles Using Replica by Prebiotics-Containing Cosmetic Serum Treatment

**Figure 3:**
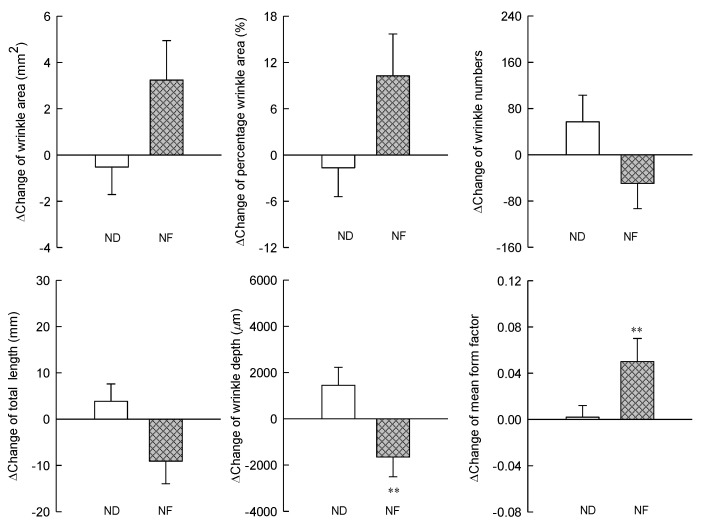
Effects of serum cosmetics containing GOS on wrinkle index from baseline after 8 weeks of treatment in healthy adults. The asterisks indicate significant differences (** *p* < 0.01) between the NF and the ND groups at the indicated week. The p of two-sided independent *t*-test method was displayed, and all data are reported as mean ± standard error of the mean. ND (control serum); NF (cosmetic serum containing GOS).

The changes in the wrinkle-related factors in the NF and ND groups before and after the serum application were measured using a replica and Visioline. The wrinkle area after the experiment increased in the NF group but was not significantly different when compared with that in the ND group. Similarly, the number, total length, and depth of wrinkles in the NF group decreased after the experiment but was not significantly different. The mean form factor, which is the average ratio of the width and length of all shadows considered wrinkles in the Visioline analysis, was significantly increased in the NF group compared with that in the ND group (Figure 3). Visioline documentation states, “This element is always between 0 (seamless circle) and 1 (seamless line). The closer the form factor is to 1, the more effective is the anti-wrinkle treatment” [21].

### 3.4. Changes in the Water Holding Capacity of the Skin Surface and TEWL by Prebiotics-Containing Cosmetic Serum Treatment

The 16S rRNA gene amplificons were subjected to MiSeq sequencing (Illumina, San Diego, CA, USA). We used a dataset of passed filter paired-end raw sequence reads and detected 15,132 operational taxonomic units (OTUs) in all groups. In total, 2810 and 3020 isolates were obtained from eight individuals in the pre-ND and pre-NF groups, respectively, and 152–991 (average, 351.3 ± 94.7) and 259–594 (average, 377.5 ± 39.5) clones were analyzed for each subject from pre-ND and pre-NF groups, respectively. *Actinobacteria*, *Firmicutes*, and *Proteobacteria* were identified as the main phyla before treatment. *Proteobacteria* accounted for 40.75% and 41.63% of all isolates in the pre-ND and pre-NF groups, respectively; *Firmicutes*, for 37.3% and 32.18%; and Actinobacteria, for 16.46% and 18.82% (Supplementary 1). *Proteobacteria* accounted for 34.37% and 37.94% of all isolates in the post-ND and post-NF groups, respectively; *Firmicutes*, for 40.43% and 34.27%; and *Actinobacteria*, for 19.56% and 20.09% (Supplementary 2). After 8 weeks of treatment, the proportion of Firmicutes increased, while that of Proteobacteria decreased. In pre-ND and post-ND groups, the main genera observed were *Staphylococcus* (22.08% and 26.33%, respectively) and *Cutibacterium* (11.46% and 13.70%, respectively).

### 3.5. Microbial Diversity Within and between the Groups

**Figure 4:**
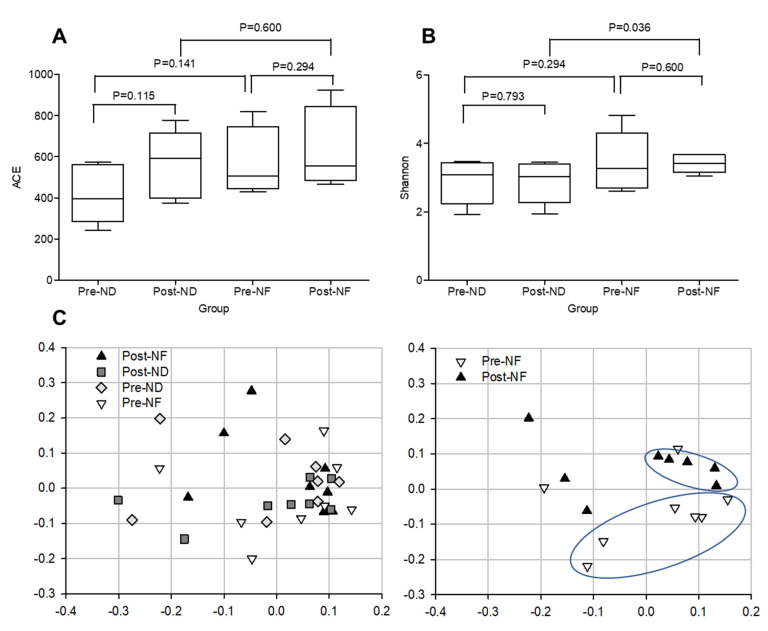
Species richness (ACE index), species diversity (Shannon index), and beta-diversity (PCoA) of species within and between groups. (**A**) Species richness (ACE index), (**B**) species diversity (Shannon index), (**C**) beta-diversity (PCoA). ND (control serum); NF (cosmetic serum containing GOS).

Alpha- and beta- diversity represent microbial diversity within or between groups, respectively. For estimation of the species richness, we calculated the number of species expected from the abundance-based coverage estimator (ACE) index. In this assessment, the species richness increased in both the post-ND and post-NF groups compared with that in pre-ND and pre-NF groups; however, no significant difference in species richness was observed between the post- and pre-treatment groups. Moreover, inter-group differences in species richness were absent (Figure 4A). The post-NF group exhibited higher species diversity than the post-ND group (Figure 4A). These results suggest that GOS-containing serum affected diversity, rather than species richness (Figure 4). Further, Shannon index was used to assess species diversity (alpha-diversity) within or between the groups (Figure 4B). Although Shannon index of the post-treatment groups showed no significant difference as compared with that of the pre-treatment groups, a significant difference in species diversity was reported between the post-ND and post-NF groups (*p* < 0.05; Figure 4B). The addition of GOS resulted in an increase in species diversity, which was significantly different from the serum base (post-ND). Our results showed that, statistically, the bacterial richness on the facial skin of the post-NF group was not significantly different compared with that of the post-ND group. However, the diversity of the bacterial communities was found to be caused by the application of GOS-containing cosmetic serum.

**Figure 5:**
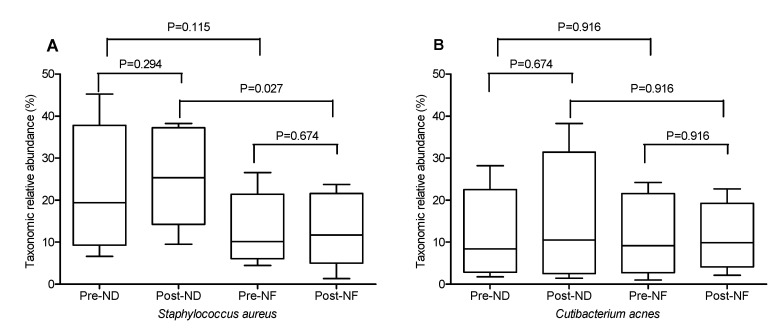
The relative abundances of *Staphylococcus aureus* (**A**) and *Cutibacterium acnes* (**B**) in the four groups. ND (control serum); NF (cosmetic serum containing GOS).

**Figure 6:**
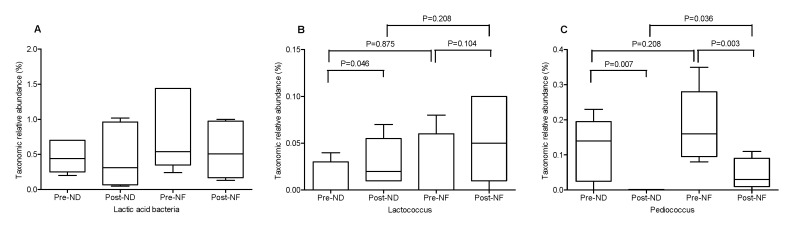
The relative abundances of Lactic acid bacteria (**A**), Lactococcus (**B**) and Pediococcus (**C**) in the four groups. ND (control serum); NF (cosmetic serum containing GOS).

Principal coordinate analysis (PCoA) was used to compare the beta-diversity profiles across different treatment groups (Figure 4C). According to PcoA (Figure 4C), the pre-NF group was mainly distributed in quadrants 3 and 4, while the post-NF group was distributed in quadrants 1 and 2. The PCoA plot indicated an obvious and a statistically significant separation between the four groups, and the center point coordinate of the ellipse was the mean value of PC1 and PC2, respectively, in the corresponding group. The ellipse was rotated to the direction of largest variation of the corresponding group. Beta set-significance analysis showed no significant differences in genera between the pre- and post-treatment groups. In the ND groups, the main genera found were *Staphylococcus* (22.08% in pre-ND and 26.33% in post-ND) and *Cutibacterium* (11.46% in pre-ND and 13.70% in post-ND) in all subjects analyzed. The main genera found in the NF groups were also *Staphylococcus* (12.518% in pre-ND and 13.19% in post-ND) and *Cutibacterium* (11.62% in pre-ND and 11.45% in post-ND). There was a significant decrease in the relative abundance of the Pediococcus genus between the pre-NF and post-NF groups (*p* < 0.05) but no significant difference between the pre-ND and post-ND groups. Moreover, there were significant species differences between the pre-NF and post-NF groups (*p* < 0.05). In the pre-ND and post-ND groups, *S*. *aureus* (22.10% and 26.27%, respectively) and *Cutibacterium acnes* (formerly called P. acnes; 11.15% and 13.48%, respectively) were most predominant (Figure 5). In particular, the abundance of *S*. *aureus*, which is known to be a pro-inflammatory bacterium, in the post-NF group was significantly decreased compared with that in the post-ND group (*p* < 0.05). Among the major genera belonging to *Firmicutes*, the abundance of *Staphylococcus* was not significantly different when compared between the pre-ND and pre-NF groups or the pre-NF and post-NF groups; however, when comparing the post-ND and post-NF groups, significant differences were found following the application of GOS-containing cosmetic serum (*p* < 0.05, Figure 5 and Supplementary 3). In contrast, no significant differences were observed in the relative abundance of the main genus belonging to the other phylum groups. *Cutibacterium* and *Enterobacteriaceae*_g populations decreased, and *Burkholderia*, *Thermoanaerobacterium*, and *Sphingomonas* populations increased in the post-NF group but were not significantly differently compared with that in the post-ND group. The relative abundance of total lactic acid bacteria in the pre-treatment groups was slightly higher than that in the post-treatment groups (Figure 6). Conversely, the relative abundance of total lactic acid bacteria in the post-NF group was higher than that in the post-ND group but the difference was not significant. This observation suggests that the serum base lowers lactic acid abundance and that GOS supplementation increases the population of lactic acid bacteria (Figure 5A). Furthermore, the *Lactococcus* population was higher in the post-treatment groups than that in the pre-treatment groups; in particular, a significant increase was observed in the post-ND group compared with that in the pre-ND group (*p* < 0.05, Figure 6B). The relative abundance of *Lactococcus* increased in the post-NF group but was not significantly different. Further, the relative abundance of Pediococcus was significantly higher in the pre-treatment groups than that in the post-treatment groups (*p* < 0.05) and in the post-NF group than that in the post-ND group (*p* < 0.05).

### 3.6. Correlation Analyses between Skin Microbiota and Skin Parameters

**Figure 7:**
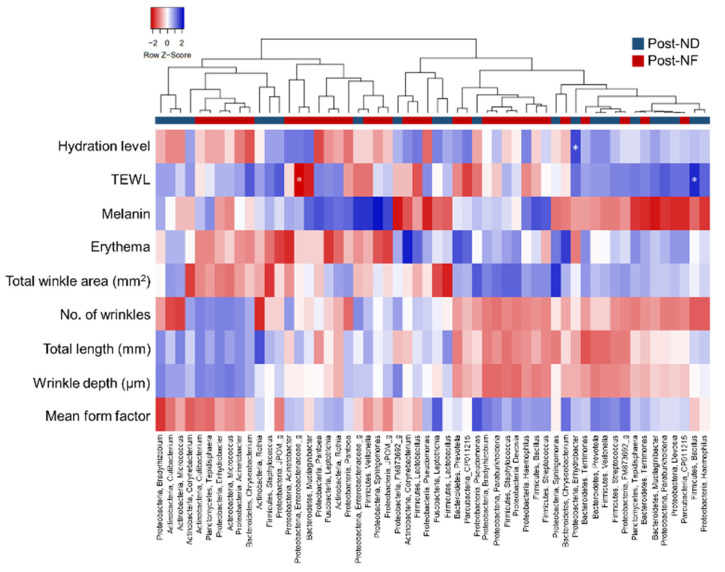
Pearson’s correlation analyses of skin microbiota with clinical parameters after treatment of serum cosmetics with/without GOS. Correlations of significant genes expression in ND and NF group with clinical parameters and skin microbiota are shown by using heat map. * Pearson’s correlation values with *p* < 0.05. ND (control serum); NF (cosmetic serum containing GOS).

Pearson’s correlation analyses were performed on the top 10 relative abundant microbial genera and skin-related parameters (Figure 7). In the ND group, water-holding capacity and *Enhydrobacter* were significantly positively correlated, compared with the other bacteria. In the NF group, however, there was a negative correlation suggesting that *Enhydrobacter* growth was inhibited by the GOS-induced growth of lactic acid bacteria. In fact, the *Enhydrobacter* population decreased from 1.53% to 0.56% following GOS-containing cosmetic serum treatment. Further, in the ND group, TEWL was a significantly positively correlated with *Bacillus*, and *Staphylococcus* was negatively correlated with the melanin and erythema indexes. Conversely, in the NF group, *Staphylococcus* was positively correlated with melanin and erythema indexes. Moreover, in the NF group, there was no significant correlation between water-holding capacity and microorganisms; but there was a negative correlation between *Enterobacteriaceae* and TEWL.

## 4. Discussion

Skin microbial communities differ from one individual to another owing to the genetic and environmental factors. Skin conditions, such as pH, temperature, and moisture content, and a variety of factors, including antibiotics, cosmetics, soaps, personal hygiene products, lifestyle, and nutrients, affect the skin microbial community and abundance. Application of probiotics and prebiotics to the skin causes specific changes to the microbial diversity and colonization, leading to reconstitution of skin microbial communities. The re-balance of skin microbiota may have a significant effect on the functional differences between healthy and damaged skin. Therefore, we evaluated the potential of GOS, a prebiotic, as a cosmetic material by measuring the changes of skin factors and skin microorganisms.

Oral ingestion of GOS has been reported to be effective in moisturizing, maintaining skin barrier function, inhibiting pigmentation [22], and improving wrinkles [23]. Unlike previous studies, GOS-containing cosmetic serum were applied to the skin in this experiment and found a significant difference in water-holding capacity and TEWL, corresponding to the skin barrier function, compared with those in the ND group (Figure 1 and Figure 2). The stratum corneum of the epidermis, the outermost layer of the skin, acts as a barrier to the harmful external environment and plays a vital role in preventing and retaining moisture in the skin [24]. The stratum corneum of the epidermis, the outermost layer of the skin, acts as a barrier to the harmful external environment and plays a vital role in preventing and retaining moisture in the skin [25], keeping the skin moist and shiny. When a lactic acid bacteria culture was applied to the skin, it maintained the pH at weakly acidic and contributed to skin hydration [24,26]. In the present study, application of GOS-containing cosmetic serum led to the growth of lactic acid bacteria among the skin microorganisms, which may have a moisturizing effect. The lactic acid bacteria such as *Streptococcus pneumoniae* (Supplementary 4) were found to be increased in the NF group compared with that in the ND group. There was no significant difference in *S. pneumoniae* levels in pre-NF and post-NF treatment group (NF) in serum treatment group containing GOS. In addition, the serum treatment group without GOS (post-ND) also showed no significant difference in *S. pneumoniae* level when compared to the post-NF group (Supplementary 5). *S. aureus* and *S. pneumoniae* are common pathogens associated with minor and invasive infections in the skin. Serum treatment containing GOS was effective in reducing *S. aureus* among pathogenic microorganisms on the skin. Additionally, oral administration of GOS increases the mRNA expression of tissue inhibitors of metalloproteinase (TIMP-1), which is an inhibitor of the collagen-degrading enzyme matrix metalloproteinase (MMP). In addition, GOS and Bifidobacterium co-administration reported a decrease in MMP-9 expression [27]. The GOS-containing serum treatment induced the proliferation of *Lactobacillus*, showing the possibility of suppressing MMP expression. The most abundant phyla identified on the skin of the subjects were *Proteobacteria*, *Firmicutes*, and *Actinobacteria* (Supplementary 2), corroborating with previous studies. *Staphylococcus* is the most common among the *Firmicutes* phylum, while *Cutibacterium* is the most abundant species among the *Actinobacteria* phylum.

Among the skin microorganisms of the subjects participating in the experiment, *Proteobacteria*, *Firmicutes*, and *Actinobacteria* were identified as the most abundant phyla (Supplementary 2). In other studies, these three phyla have been reported to be the most prevalent on the skin [28,29]. *Staphylococcus* is the most common among *Firmicutes* phylum, while actinobacteria is the most abundant species among *Cutibacterium*. The facial skin samples from the pre-treatment groups harbored fewer species than those from the post-treatment groups. Moreover, no statistically significant difference was observed in the bacterial richness on the facial skin of the post-NF group compared with the post-ND group; however, the diversity of the bacterial communities was found to be caused by the application of the GOS-containing cosmetic serum (Figure 4). GOS treatment was expected to induce higher diversity than the serum base treatment because GOS as a prebiotic enhances environmental diversity of the skin microbes. Prebiotics are “fertilizers” or “foods” with components that selectively promote the growth of these essential microorganisms, thereby potentially improving the host health [30]. *S. aureus* was the most common species found on the skin of the subjects, as shown in Supplementary 3 and Figure 5, and has been recognized as a crucial cause of skin infection [31]. *Staphylococcus* is relatively common on healthy skin but in one-third of the population the presence of *S. aureus* is considered a significant risk factor for future infections [32]. *S. aureus* weakens the skin barrier and activates deleterious host immune responses. For instance, *S*. *aureus* produces proteases that are capable of penetrating the dermis of patients or mice with atopy disease (AD) and deleterious mutations in the filaggrin gene [33,34]. In fact, in AD, abundance of *S*. *aureus* was associated with immune dysfunctions, including T helper cell 2 lymphocyte asymmetry, reduced antimicrobial peptides (AMPs), exacerbated allergic reactions, and destruction of the skin barrier [35]. *S. aureus* increases the production of type 2 cytokines, such as thymic stromal lymphopoietin, interleukin (IL)-4, and IL-13 [33]. Moreover, several other molecules produced by *S. aureus* also induce skin inflammation and exacerbate AD. In particular, α-toxin degrades keratinocytes in the presence of type 2 cytokines. All these factors contribute to local inflammation and further affect the skin barrier, leading to the exacerbation of skin diseases such as AD [36]. However, other bacterial species found on normal skin seem to help maintain immune homeostasis [37]. *S. epidermidis* present on the skin of healthy individuals may reduce inflammation [38], improve the development of skin T cells, and promote the expression of AMPs [39]. 

Skin sites can be classified based on their physiological properties as sebaceous (oily), moist, or dry and can affect the distribution of the skin microbiota [2]. *Enhydrobacter*, *Corynebacterium*, and *Staphylococcus* are predominant at the moist sites. In the ND group, water-holding capacity was positively correlated with these strains (Figure 7). Moreover, TEWL was positively correlated with the relative abundance of *S. aureus* but was not significantly different (Figure 7). In the human AD epidermis, the relative abundance of *S. aureus* and *S. epidermis* is increased while that of *Propionibacterium* decreased relative to other genera (*Streptococcus*, *Acinetobacter*, *Corynebacterium*, and *Prevotella*). Furthermore, in this study, *Bacillus* and TEWL were significantly positively correlated in the ND group. Bacillus is a genus belonging to Firmicutes and is found on normal skin [40]. Further, increased colonization of *Enterobacteriaceae* is observed in adult and infant populations with AD [41,42]. In fact, in the case of AD, TEWL is reduced, demonstrating a negative correlation between *Enterobacteriaceae* and TEWL.

In our previous study [22,43], GOS were shown to inhibit the production of pro-inflammatory agents IL-6, IL-8, and IL-12; tumor necrosis factor-α; and prostaglandin E2 in UV-irradiated HaCaT cells and hairless mice. The increase in the population of lactic acid bacteria and inhibition of inflammation-producing substances by GOS may have reduced the population of *S. aureus* and production of pro-inflammatory substances. The application of cosmetics containing prebiotics from certain plant extracts is effective in inhibiting the growth of the inflammation-causing bacterium but not that of coagulase-negative *Staphylococci* [8]. The application of prebiotics is far superior to that of antimicrobial cosmetics, which use antibiotics or antibacterial agents to unselectively reduce bacterial growth [44]. Moreover, prebiotics such as GOS improve the host health by selectively stimulating the growth of beneficial bacterial species such as *Bifidobacteria* and *Lactobacilli*. Additionally, various lactic acid bacteria, including *Lactobacillus*, *Lactococcus*, *Pediococcus*, *Propionibacterium*, *Leuconostoc*, and *Carnobacterium* have been reported to use prebiotics for the production of bacteriocins [45,46]. Oh et al. [46] reported a clinical test which revealed that bacteriocin HY 449 from *Lactococcus* spp. regulates skin inflammation and acne and inhibits the growth of inflammatory skin bacteria, such as *S. aureus*, *Streptococcus pyogenes*, and *P*. *acnes*. The production of bacteriocin, owing to GOS-induced lactic acid secretion, may also reduce the number of harmful microorganisms on the skin.

## 5. Conclusions

In conclusion, a randomized controlled trial was used to evaluate skin-related parameters in healthy adults who treated with GOS-containing cosmetic serum for 8 weeks. This study supports the principal concept that the application of GOS-containing serum can be used for the management of the composition and diversity of the human skin microbiota. Treatment with GOS-containing cosmetic serum is effective in improving various skin parameters and the population of beneficial skin microbes. However, since the analysis of the skin microbial community was performed in a small number of subjects, additional experiments are needed to investigate the correlation between the application of GOS and the growth of harmful microorganisms such as *Streptococcus* spp. at various ages and subjects.

## Supplementary Materials

The following are available online at https://www.mdpi.com/2075-4426/10/3/91/s1, Figure S1: The relative abundance of the most prevalent bacterial groups associated with each microenvironment depicted for all the subjects investigated. Superscripts indicate phylum; Figure S2: The relative abundance of the most prevalent bacterial groups in each group; Table S1: The relative abundance of the main genera of bacteria in post-treatment groups (post-ND and post-NF); Table S2: The relative abundance of the main species of bacteria in post-treatment groups (post-ND and post-NF).
