# Supplementary material for: Changes in the Diversity of Human Skin Microbiota to Cosmetic Serum Containing Prebiotics: Results from a Randomized Controlled Trial
Source: J Pers Med. 2020 Aug 17;10(3):91. doi: 10.3390/jpm10030091 (PMC7564969; doi:10.3390/jpm10030091)
Supplement: Supplementary file 1 [file jpm-10-00091-s001.pdf]

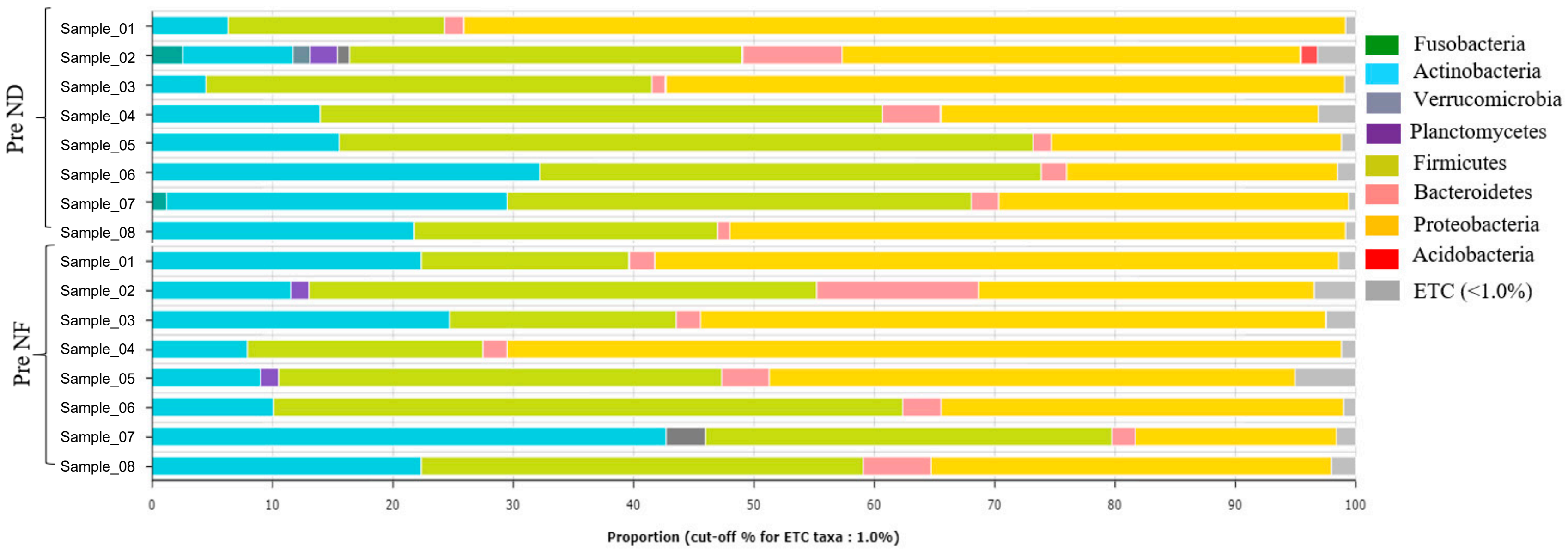

Sup. 1. The relative abundance of the most prevalent bacterial groups associated with each microenvironment depicted for all the subjects investigated. Superscripts indicate phylum.

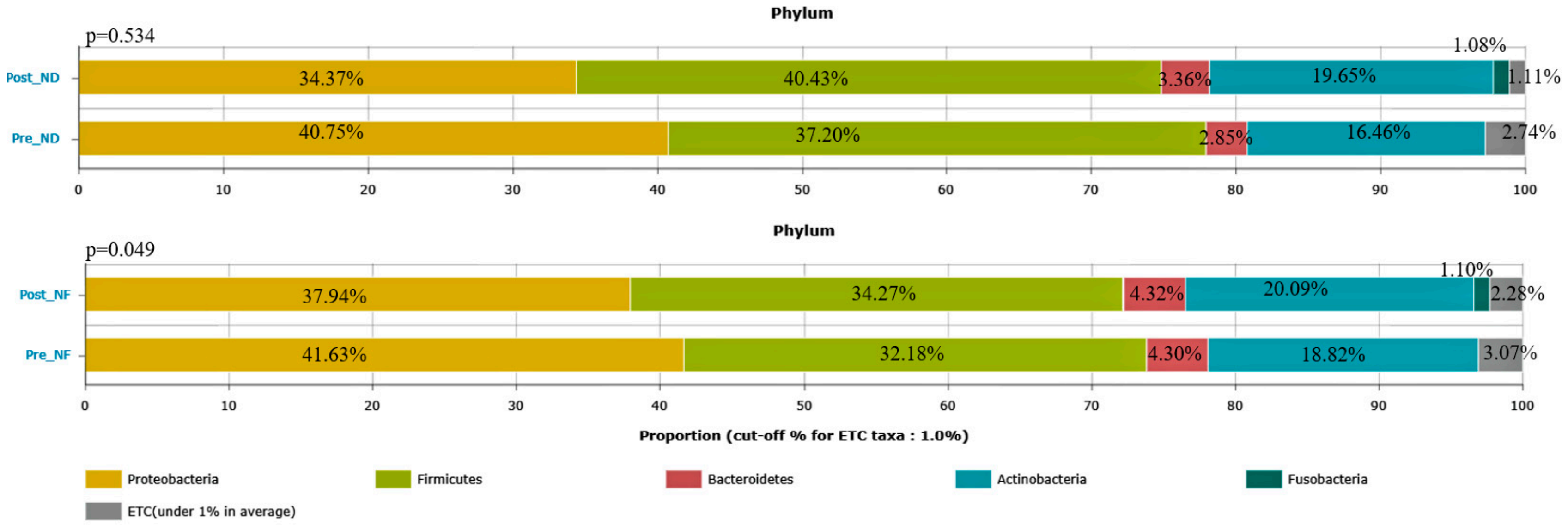

Sup. 2. The relative abundance of the most prevalent bacterial groups in each group.

Sup. 3. The relative abundance of the main genera of bacteria in post-treatment groups (post-ND and post-NF)

Phylum	Genus	Group	Abundance (%)	*P* value	Phylum	Genus	Group	Abundance (%)	*P* value
Firmicutes	*Staphylococcus**	Post-ND	26.32 ± 12.06	0.027	Proteobacteria	*Burkholderia*	Post-ND	7.10 ± 3.01	0.462
		Post-NF	13.18 ± 8.85				Post-NF	8.42 ± 5.56	
	Streptococcus	Post-ND	1.63 ± 1.08	0.208		*Sphingomonas*	Post-ND	3.29 ± 1.01	0.462
		Post-NF	4.25 ± 4.29				Post-NF	3.83 ± 2.02	
	Thermoanaerobacterium	Post-ND	3.74 ± 2.05	0.074		*Enterobacteriaceae_g*	Post-ND	4.11 ± 2.61	0.753
		Post-NF	6.67 ± 4.27				Post-NF	1.06 ± 1.02	
	Anaerococcus	Post-ND	2.53 ± 2.07	0.916	Actinobacteria	*Cutibacterium*	Post-ND	13.69 ± 12.27	0.916
		Post-NF	2.33 ± 2.57				Post-NF	11.44 ± 8.17	
Bacteroidetes	*Prevotella*	Post-ND	1.60 ± 1.17	1.00		*Corynebacterium*	Post-ND	1.54 ± 0.79	0.674
		Post-NF	1.72 ± 2.24				Post-NF	0.93 ± 0.51	
						Lawsonella	Post-ND	1.77 ± 2.76	0.600
							Post-NF	3.08 ± 2.05	

The asterisks indicate significant differences (*$p < 0.05$) between the NF and the ND groups in the indicated Genus.

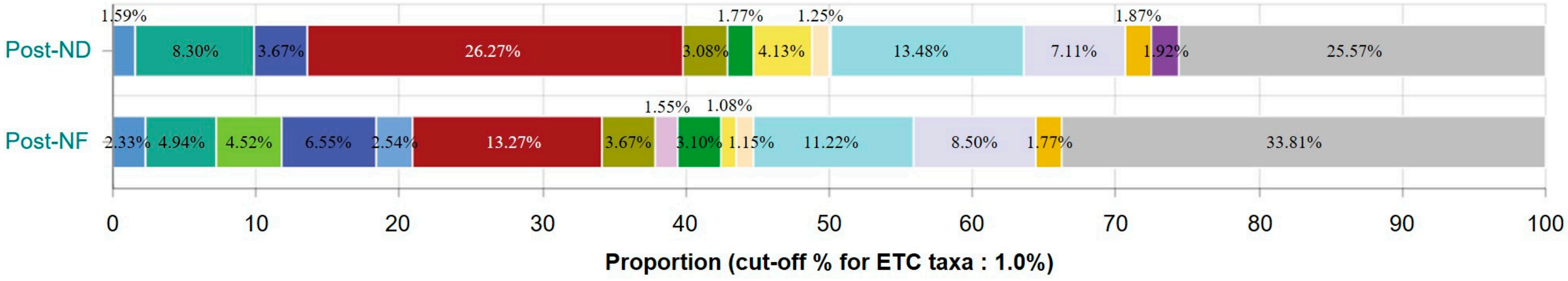

	Microbial number (counts/DNA)			Microbial number (counts/DNA)	
	Post-ND	Post-NF		Post-ND	Post-NF
Caulobacteraceae JPOM_g PAC001012_s	172.16	367.23	*Neisseriaceae* FM873692_g FM873692_s	898.67	778.59
Xanthomonas citri group	-	712.39	*Thermoanaerobacterium thermosulfurigenes* group	397.36	1032.340
Streptococcus pneumoniae group	-	400.33	*Staphylococcus aureus* group	2844.35	2091.47
Sphingomonas pruni group	333.48	578.42	*Neisseria perflava* group	-	244.29
Lawsonella clevelandensis	191.64	488.59	*Enterobacteriaceae* group	477.17	170.22
Enhydrobacter aerosaccus group	146.75	181.55	*Cutibacterium acnes* group	1459.53	1768.37
Burkholderia cepacia group	769.83	1339.67	*Anaerococcus nagyae* group	202.47	278.97
Dolosigranulum pigrum group	207.89	-	ETC (under 1% in average)	2768.56	5328.75

Sup. 4. The relative abundance of the main species of bacteria in post-treatment groups (post-ND and post-NF)

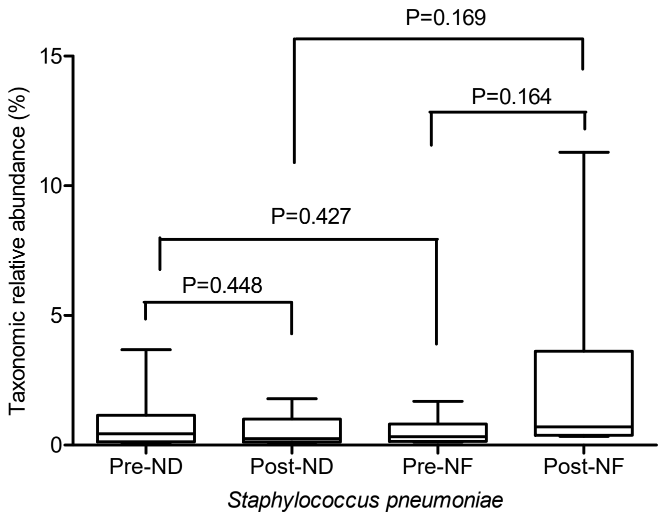

Sup. 5. *Staphylococcus pneumoniae* difference within and between groups. ND (control serum); NF (cosmetic serum containing GOS).
